# Expert consensus on sustainable employability of older workers: research, practice, and policy dimensions

**DOI:** 10.3389/fpsyg.2026.1903399

**Published:** 2026-09-14

**Authors:** Luiza de Souza Coroa Contage, Lucia Helena de Freitas Pinho França

**Affiliations:** Postgraduate Program in Psychology, Universidade Salgado de Oliveira, Niterói, Brazil

**Keywords:** aging, Delphi method, expert consensus, older workers, sustainable employability

## Abstract

**Introduction:**

Sustainable employability has been recognized as a central construct for understanding workers’ continued participation in the labor market throughout the life course, particularly in the context of population aging, technological transformations, and changing work arrangements. Despite growing research in this field, conceptual fragmentation and a lack of consensus persist regarding the key factors, organizational practices, and public policies associated with the construct. Accordingly, this study aimed to identify factors, organizational practices, public policies, and research dimensions related to the sustainable employability of older workers from the perspective of international academic experts.

**Methods:**

A sequential exploratory mixed-method Delphi design was adopted. Fourteen experts from different countries participated in the first Delphi round, with 12 completing the second round and 9 completing the final round. The first qualitative stage was conducted as a structured elicitation exercise and organized participants’ responses into five analytical domains: the definition of sustainable employability, organizational practices, public policies, individual factors, and research dimensions. In the subsequent rounds, the items derived from these domains were quantitatively evaluated for expert agreement.

**Results:**

High levels of consensus were observed regarding physical and mental health, lifelong learning, healthy work environments, organizational practices to combat ageism, workplace flexibility, and the integration of health, social security, and employment policies. Additional dimensions, including meaning of work, career adaptability, work–life balance, and support networks, also achieved the predefined consensus criteria in the final Delphi round. Divergence remained primarily for selected policy-related dimensions, particularly tax incentives for companies and funding for age-friendly workplace adaptations.

**Discussion:**

The findings support the conceptualization of sustainable employability as a multilevel and contextual construct encompassing individual resources, organizational practices, and institutional policies. This study contributes to the conceptual advancement of the field by providing expert-based evidence that clarifies the construct and identifies dimensions that achieved expert consensus, thereby informing future research, organizational practice, and public policy.

## 1 Introduction

Sustainable employability has emerged as a central construct for understanding workers’ continued participation in the labor market throughout their life course. It refers to individuals’ capacity to remain employable and functionally active throughout their working lives, under conditions that promote health, wellbeing, productivity, and meaning at work (Van der Klink et al., 2016). Unlike traditional approaches that focus mainly on obtaining and maintaining employment, this perspective broadens employability by incorporating, in addition to individual factors, organizational, social, and governmental aspects that influence the continuity of labor participation (Vermeeren and Van der Heijden, 2022).

The relevance of this construct is manifested at different levels. At the individual level, work is associated with financial security, a sense of purpose, the development of social networks, and the promotion of health and wellbeing (Schuring et al., 2015). At the organizational level, the sustainability of performance depends on the ability to rely on workers who can contribute continuously throughout their lives (Fleuren et al., 2020). At the governmental/social levels, this sustained participation is directly linked to economic balance and the sustainability of social protection systems, especially through the retention of older workers in the labor market (Alemayehu and Warner, 2004).

However, this scenario is shaped by structural transformations that make sustainable employability simultaneously more necessary and more challenging. Population aging (Fisher et al., 2016) and its consequences for the sustainability of pension systems (Nagarajan and Sixsmith, 2023), the shortage of skilled labor (Muzam, 2023), rapid technological changes (Daher and Ziade, 2023), and the reconfiguration of work arrangements (Van der Klink et al., 2016) impose new demands on workers and organizations, requiring continuous updating of skills and adaptation to increasingly dynamic contexts (Cascio and Montealegre, 2016; Van Dam et al., 2017). Therefore, a deeper scientific understanding of sustainable employability and its determinants is essential to sustain individual labor participation, organizational functioning, and social wellbeing.

Although research on sustainable employability has expanded considerably, important conceptual challenges remain. Current evidence indicates that the construct is still characterized by theoretical fragmentation, with individual, organizational, and policy-related dimensions often being examined separately rather than within an integrated framework (De Lange et al., 2022; De Vos et al., 2020). Although the definition proposed by Van der Klink et al. (2016) remains the most widely adopted, it has been criticized for not fully capturing the complexity of sustainable employability and the dynamic interaction between individual, organizational, and contextual factors (Fleuren et al., 2016; Fleuren et al., 2020). Consequently, there is still no clear consensus regarding the core dimensions that define sustainable employability.

Recent approaches have brought employability closer to the field of sustainable careers, understanding professional trajectories as dynamic and multidimensional processes influenced by personal resources, working conditions, and institutional contexts (Contage and França, 2024; De Vos et al., 2020; Donald et al., 2024; Neupane et al., 2022). The concept of sustainable careers refers to the continuity of professional trajectories, simultaneously considering dimensions such as age, health, happiness, productivity, and meaning of work (De Vos et al., 2020). Unlike traditional perspectives that focus solely on job stability or hierarchical progression, the sustainable career perspective emphasizes context and the dimensions mentioned above throughout the life course.

It is important to distinguish between the concepts of sustainable work, sustainable careers, and sustainable employability. In this context, sustainable employability emerges as a fundamental component of sustainable careers (Van der Klink et al., 2016). Sustainable work refers to working conditions that allow individuals to perform their activities in a healthy, safe, meaningful, and wellbeing-compatible manner over time, considering both organizational, social, and individual aspects (Fleuren et al., 2020). Sustainable careers, in turn, constitute a broader construct, related to the continuity, adaptability, and quality of professional experiences over time (Van der Heijden and De Vos, 2015; De Vos et al., 2020). Sustainable employability, on the other hand, refers more specifically to the capacity to maintain continuous, healthy, and productive participation in the labor market throughout working life (Van der Klink et al., 2016). In this sense, sustainable employability can be understood as a central element in sustaining careers across the life cycle, influenced by the conditions of sustainable work offered to workers.

Although sustainable employability is relevant throughout the working life, it becomes particularly important among older workers, who are more likely to experience changes in health, work ability, retirement decision-making, and exposure to age-related stereotypes and discrimination (Fisher et al., 2016; Nilsson, 2020; Van der Klink et al., 2016). At the same time, rapid technological changes, evolving work arrangements, and demographic aging require older workers to continuously adapt their knowledge and skills while maintaining their wellbeing and productivity (Cascio and Montealegre, 2016; Van Dam et al., 2017). Consequently, sustainable employability in later working life depends on the articulation of individual resources, organizational practices, and supportive public policies, making older workers a particularly relevant group for advancing the conceptual understanding of this construct (De Vos et al., 2020; Nilsson, 2020).

Moreover, recent models, such as the Swage Model (Nilsson, 2020), have emphasized that sustainable participation in work depends on the interaction among health, competencies, motivation, and organizational conditions throughout the life course. Recent studies reinforce the idea that the sustainability of professional trajectories depends on continuous processes of generating, maintaining, and renewing resources (Annosi et al., 2025; Jabeen et al., 2022). Elements such as lifelong learning (Le et al., 2023), adequate working conditions (Contage et al., 2026; Nilsson et al., 2023), organizational support (Gürbüz et al., 2024), meaning of work (Pransky et al., 2016), and inclusive public policies (Grau-del-Valle et al., 2025) have been identified as fundamental for sustaining labor market participation throughout the life course.

Complementing this perspective, the Ability–Motivation–Opportunity (AMO) framework proposes that sustainable work participation results from the interaction between workers’ abilities, motivation, and the opportunities provided by organizations (Le Blanc et al., 2017). Although originally developed in the human resource management literature, the AMO framework offers a useful theoretical lens for understanding how individual resources and organizational conditions influence sustainable employability. Together, the Swage and AMO models reinforce the idea that sustainable employability is a multidimensional construct shaped by the dynamic interaction between individual capabilities and contextual factors across the working life course.

Despite these theoretical advances, the literature still exhibits considerable conceptual heterogeneity and thematic dispersion, with different studies prioritizing distinct dimensions without reaching a clear consensus regarding the key components of sustainable employability (Mo et al., 2024). This lack of convergence poses a significant obstacle to the field’s development, as it hinders the accumulation of cumulative knowledge and the formulation of more structured research agendas (Cheng, 2022). In emerging multidimensional fields such as sustainable employability, identifying dimensions considered relevant by experts is an essential step toward theoretical and empirical advancement (Van der Waldt, 2024).

Thus, expert consensus methods prove appropriate, as they allow the integration of diverse perspectives, the systematization of existing knowledge, and the identification of relevant gaps (Keeney et al., 2011). Among these methods, the Delphi technique stands out for enabling the structured construction of consensus through successive rounds of evaluation, reducing the influence of biases and promoting the convergence of informed judgments (Jain et al., 2024; Majka, 2024).

A key challenge is not only to clarify the concept but also to identify the dimensions considered most relevant by experts necessary to achieve sustainable employability in the context of aging. Therefore, it is essential to use a strategy that integrates the knowledge, experience, and expertise of specialists to advance not only in the conceptual consolidation of the construct, but also in the development of future studies and in innovative proposals for sustainable employability that may contribute to addressing the balance of pension systems, both in aging countries and in emerging countries that are undergoing aging, supporting public policies, organizational practices, as well as the promotion of more sustainable work trajectories (Walker, 2002).

Although previous studies have identified several determinants of sustainable employability, there is still limited consensus regarding how these determinants should be conceptually organized across individual, organizational, and policy levels. Consequently, this study seeks not only to identify relevant factors but also to clarify how experts conceptualize sustainable employability among older workers and identify dimensions relevant to research, organizational practice, and public policy. Thus, this study aimed to identify the main factors, practices, policies, and research priorities related to the sustainable employability of older workers, from the perspective of academic experts in the field, using the Delphi technique as a methodological strategy. Specifically, this study sought to answer the following research questions: (1) How do experts conceptualize sustainable employability among older workers? (2) Which individual factors are considered essential for sustainable employability? (3) Which organizational practices and public policies are considered priorities for promoting sustainable employability? (4) Which research priorities should guide future investigations in this field? and (5) To what extent do experts reach consensus regarding these dimensions across successive Delphi rounds?

## 2 Method

This study employed a sequential exploratory mixed-method Delphi design, in which qualitative findings from the first round informed the quantitative consensus assessment conducted in subsequent rounds (Gil, 2025; Minayo and Guerriero, 2014). The online Delphi technique was used to develop this study, which is characterized by its ability to generate consensus on a given topic (Giannarou and Zervas, 2014). The qualitative phase was used to generate themes and questionnaire items, whereas the quantitative phases aimed to establish expert consensus regarding these items.

### 2.1 Participants

For participant selection, academic experts with demonstrated scientific expertise in sustainable employability, aging, work, or closely related fields were considered, aiming to obtain a qualified consensus among national and international researchers. Potential experts were identified through a structured search conducted in the Scopus and Web of Science databases between 2022 and 2025. The search strategy used the Boolean expression (“older workers” OR aging OR “extended working life”) AND (employability OR employment OR “age diversity”). After duplicate records were removed, the publication profiles of the retrieved researchers were manually screened to assess their scientific contribution to the field and determine their eligibility for the study, resulting in the preliminary identification of 40 potential experts.

Experts were defined as researchers with demonstrated scientific expertise in sustainable employability, aging, work, retirement, or closely related fields. Eligibility criteria included: (a) at least 3 years since completion of the PhD; (b) an active research agenda related to the topic, evidenced by participation in research projects; and (c) peer-reviewed publications indexed in Scopus or Web of Science addressing sustainable employability, aging, work, retirement, or related themes. These criteria were adopted to ensure that participants possessed both theoretical knowledge and research experience relevant to the objectives of the Delphi study. After applying the eligibility criteria, 30 researchers were considered eligible and were formally invited to participate. Of these, 14 agreed to participate and fully completed the first Delphi round, corresponding to a response rate of 46.7%.

A total of 14 experts participated in the first round of the Delphi study (Delphi 1). Throughout the subsequent rounds, a progressive reduction of the panel was observed, with 12 participants in the second round (Delphi 2) and 9 in the third round (Delphi 3). Of the 12 experts who completed Delphi 2, nine completed Delphi 3. The final panel comprised experts from seven countries: Brazil (*n* = 2), England (*n* = 1), Chile (*n* = 1), Mexico (*n* = 1), Colombia (*n* = 1), New Zealand (*n* = 1), and the Netherlands (*n* = 1). Thus, despite the attrition observed across rounds, the final panel retained geographical diversity across Latin American, European, and Oceanian contexts. This reduction is commonly observed in Delphi studies involving multiple rounds (Powell, 2003). Nevertheless, panel attrition may influence the stability of consensus and should therefore be considered when interpreting the findings. Despite this limitation, the remaining panel continued to comprise researchers with extensive expertise in the field (Table 1).

**Table 1:** Participants’ sociodemographic and professional characteristics.

Variable	Category	*n*	%
Sex	Female	10	71.4
	Male	4	28.6
Age group (years)	35–54	8	57.1
	55 or more	6	42.9
Experience in the field	3–10 years	5	35.7
	10–20 years	7	50.0
	20 years or more	2	14.3
Geographic scope	Latin America	7	50.0
	Central America	1	7.1
	Europe	5	35.7
	Oceania	1	7.1

Percentages calculated based on the total number of participants in the first round (*n* = 14).

To ensure participant adherence and continuity across rounds, follow-up contacts (reminders) were conducted with the invited experts. In the first round, four reminders were sent at intervals of two to 3 months. In the second round, three reminders were conducted at the same interval. In the third round, five reminders were sent at approximately one-month intervals to increase the response rate and minimize sample loss throughout the Delphi process.

At baseline, the 14 participants were researchers with established experience from different countries, including Germany, Brazil, Chile, Colombia, Mexico, New Zealand, the Netherlands and the United Kingdom, broadening the range of perspectives considered in the consensus process and contributing to the diversity of perspectives and the robustness of the consensus achieved. The participants’ ages ranged from 35 to 68 years, with a mean of 51.6 years (SD = 8.3), indicating a group predominantly composed of professionals at an advanced career stage. A predominance of female participants was observed (71.4%), with male participation corresponding to 28.6% of the sample (Table 1).

Regarding professional experience in the field, most experts had well-established trajectories: 50% reported between 10 and 20 years of experience, 35.7% had up to 10 years, and 14.3% had more than 20 years. These data indicate that the panel was predominantly composed of experts with significant and consolidated experience in the field.

### 2.2 Data collection procedures

Invitations were sent by email to 30 previously selected professionals, accompanied by a letter of introduction from the researcher that included a brief description of the project objectives and a request for collaboration in completing the questionnaire, with an initial response deadline of 15 days. Among the invited experts, 14 agreed to participate and fully responded to the first round of the study. Those who expressed interest confirmed their participation via Google Forms by validating the Informed Consent Form (ICF) and completing the study’s first round. Consent was formalized after participants had full knowledge of the objectives, procedures, and ethical conditions of the research. This procedure is in accordance with the Resolutions of the Brazilian National Health Council (CNS) No. 466/2012 and No. 510/2016, which regulate research involving human subjects in the Brazilian context.

The approach to the experts was intentional and individualized, taking into account the participants’ academic expertise and scientific output in employability, aging, and work. After selection, the experts were individually contacted via institutional email and received an invitation letter outlining the study objectives, research justification, estimated participation time, and clarifications on the dynamics of the Delphi technique.

Considering the international nature of the panel, the data collection instruments and invitations sent to the experts were primarily developed in English. Most participants responded to the questionnaires in English. However, some Brazilian experts chose to respond in Portuguese, and their responses were subsequently translated into English for analytical standardization. The translation procedures sought to preserve the semantic meaning of the original responses, considering the construct’s conceptual specifics.

The Delphi process spanned approximately 11 months, encompassing participant recruitment, the completion of three Delphi rounds, and follow-up reminders. The relatively long intervals between rounds were primarily due to the difficulty of coordinating the participation of internationally recognized experts located in different countries and time zones. Maintaining participant engagement required repeated individualized follow-up contacts, which extended the duration of the Delphi process. Although these intervals may have contributed to panel attrition, maintaining a panel of internationally recognized experts was considered more important than shortening the intervals at the expense of losing participants with substantial expertise. This challenge is common in Delphi studies involving highly specialized international panels (Keeney et al., 2011).

In the initial stage (Delphi 1), a semi-structured online questionnaire was distributed via email, comprising two sections. The first included the Informed Consent Form (ICF) and sociodemographic questions (age, sex, country of residence, years since graduation, and professional experience). The second consisted of five open-ended questions addressing the definition of sustainable employability, individual factors, organizational practices, public policies, and research priorities concerning the sustainable employability of older workers. To enhance response rates and ensure participant adherence, four reminders were sent during this round at approximately two- to three-month intervals. The five open-ended questions were intentionally designed to elicit expert perspectives on conceptually distinct domains of sustainable employability. Consequently, the qualitative analysis sought to identify the principal themes within each domain rather than derive an entirely inductive category structure.

Based on the qualitative analysis conducted in the first round, the quantitative questionnaire for the second stage was developed. In Delphi 2, participants received an individualized invitation by email to complete the questionnaire incorporating the themes identified in the qualitative analysis of Round 1. Because Round 1 consisted exclusively of open-ended questions, no quantitative controlled feedback was provided at this transition. Participants rated each item using a five-point Likert scale (1 = Strongly Disagree to 5 = Strongly Agree) and were invited to justify their ratings and provide comments on each item, allowing qualitative feedback to complement the quantitative consensus process. To promote sustained engagement, three reminders were sent at approximately two- to three-month intervals.

After completion of Delphi 2, responses were compiled and analyzed using the predefined consensus criteria (median, interquartile range, and percentage agreement). Before Delphi 3, participants received controlled quantitative feedback summarizing the results of Delphi 2, including the group median, the distribution of responses, and anonymized comments. They were then invited to reassess the items that had not reached consensus and to evaluate new items suggested by the panel. The controlled feedback report provided before Delphi 3 is presented in Supplementary Material S5. The third-round questionnaire therefore consisted of the items that had not reached consensus in Delphi 2, together with additional items generated from participants’ suggestions. Five reminders were sent at approximately one-month intervals to maximize panel retention until the completion of the study. The five open-ended questions used in Round 1, the traceability of qualitative findings to the Round 2 items, the questionnaires administered in Rounds 2 and 3, and the controlled feedback provided between Rounds 2 and 3 are provided in Supplementary Materials S1–S5.

### 2.3 Data analysis procedures

Data analysis was conducted according to the different stages of the Delphi process. In the first round, participants’ open-ended responses were analyzed using the Requalify.ai platform (version 0.1) (Martins et al., 2024), which applies Natural Language Processing (NLP) techniques to identify lexical co-occurrence patterns and recurrent semantic structures within textual data. The software generated preliminary thematic categories based on the semantic relationships identified across participants’ responses.

The qualitative analysis followed a two-step procedure. First, textual data was processed using NLP techniques to identify lexical co-occurrence patterns and recurrent semantic structures across participants’ responses. The software generated preliminary thematic categories based on the semantic relationships identified within the dataset. Second, these preliminary categories were independently reviewed and interpreted by the first and last authors, who jointly evaluated their conceptual coherence, relevance to the study objectives, and representativeness of the participants’ responses. The final thematic structure was established through consensus between the researchers. Formal intercoder agreement statistics were not calculated because the objective was to achieve interpretative consensus regarding the conceptual organization of the themes rather than to quantify coding agreement.

The objective of using Requalify.ai was not to automate qualitative coding, but rather to support the systematic identification of lexical co-occurrence patterns and recurrent semantic structures within the dataset. Therefore, the software functioned as a decision-support tool for organizing and synthesizing textual data, whereas the interpretation of the categories, thematic classifications, and all analytical decisions remained the responsibility of the research team. Furthermore, the qualitative phase of the first Delphi round was intended to identify recurring themes and generate questionnaire items for the subsequent Delphi rounds rather than to develop a fully interpretative qualitative theory. Consequently, NLP-assisted procedures were considered appropriate for supporting thematic exploration while preserving researcher oversight throughout the analytical process. This approach is consistent with the broader use of computer-assisted qualitative data analysis software (CAQDAS), which supports data organization and pattern identification while maintaining researcher responsibility for interpretation and validation (Silver and Lewins, 2014; O’Kane et al., 2023; Paulus, 2023).

These preliminary categories were subsequently reviewed and interpreted by the first and last authors, who jointly evaluated their conceptual coherence, relevance to the study objectives, and representativeness of the participants’ responses. The final thematic structure was established through consensus between the researchers. Formal intercoder agreement statistics were not calculated because the objective was to achieve interpretative consensus regarding the conceptual organization of the themes rather than to quantify coding agreement. Thus, Requalify.ai served as a decision-support tool for organizing and synthesizing textual data, whereas the interpretation of the categories and all analytical decisions remained the responsibility of the research team.

The final categories derived from the qualitative analysis constituted the basis for developing the questionnaire used in the second Delphi round. Each category was converted into one or more questionnaire statements representing the central ideas expressed by the experts. When a category encompassed multiple related concepts, separate items were created to capture each specific dimension while preserving the conceptual meaning of the original responses and ensuring their suitability for quantitative evaluation. The progression from the qualitative themes identified in the first Delphi round to the questionnaire items developed for the second round is presented in Supplementary Table S2.

In the second and third Delphi rounds, the objective was to establish the level of consensus among experts regarding the questionnaire items generated from the qualitative analysis. Given the absence of universally accepted criteria for defining consensus in Delphi studies (Staykova, 2019), a combination of indicators frequently recommended in the literature was adopted (Van Houwelingen et al., 2016). Consensus was considered to have been achieved when an item simultaneously met the following criteria: (a) median (Md) ≥ 4, indicating a high level of agreement; (b) interquartile range (IQR) ≤ 1.0, indicating low variability in responses; and (c) percentage agreement ≥75%, calculated as the proportion of experts selecting responses in the upper categories of the Likert scale (ratings of 4 = Agree or 5 = Strongly Agree) (Diamond et al., 2014; Nasa et al., 2021; Price et al., 2020). These criteria were established *a priori*, before data analysis. Items that did not meet one or more of these criteria were retained for reassessment in the subsequent Delphi round. The interpretation of the results considered these three indicators jointly to distinguish items that achieved consensus from those requiring further evaluation. The combination of these three indicators was intended to minimize the influence of any single statistic when determining consensus. Google Forms was used to administer the questionnaires, and statistical analyses were performed using Microsoft Excel and JASP for Windows (version 0.18).

## 3 Results

### 3.1 Results of the first round (Delphi 1)

The first Delphi round was designed as a structured elicitation exercise. The qualitative analysis organized participants’ responses into five analytical domains corresponding to the topics explored in the interview guide while identifying the principal themes expressed within each domain. The experts’ open-ended responses were analyzed using lexical co-occurrence analysis, conducted with the support of the Requalify.ai platform (Martins et al., 2024), thereby identifying patterns of association between terms and the underlying semantic structure of participants’ discourse. The qualitative analysis organized participants’ responses into five analytical domains corresponding to the topics explored in the Round 1 questionnaire: (1) definition of sustainable employability, (2) organizational practices, (3) public policies, (4) individual factors, and (5) research priorities.

Some themes, particularly flexibility and lifelong learning, emerged across more than one analytical domain. Rather than representing mutually exclusive categories, these themes were classified according to the primary context in which they were discussed by the experts. This approach acknowledges the inherently multilevel nature of sustainable employability, in which individual, organizational, and policy-related dimensions are closely interconnected. Accordingly, the domains presented in the subsequent Delphi rounds reflect the context in which the experts originally discussed these themes during the structured elicitation, rather than *a priori* theoretical classification.

*Axis 1—Definition of sustainable employability.*

The analysis showed that the concept is organized around central terms such as “sustainable employability (as a continuous ability)”, indicating that sustainable employability is viewed as an individual’s continuous capacity to remain active and satisfied in the labor market, adapting to changes and ensuring that employment is secure and rewarding. As illustrated in the participants’ statements: “Sustainable employability is enabling individuals to be employed for as long and as continuously as possible, making reasonable adjustments to meet individuals’ needs (P3)”; “It implies the possibility of continuing to be employable throughout life, especially in relation to possible age-related biases. In this sense, employability needs to encompass health, happiness, and productivity throughout life (P4)”; and “Sustainable employability, for me, means a person’s continuous ability to remain effectively in the labor markets of their interest throughout their working life, while maintaining their wellbeing and adapting to constantly changing circumstances. This goes beyond simply having a job; it is about the ongoing ability to secure and maintain satisfying and productive work. People can participate in the workforce in suitable employment for as long as they wish” (P8).

Another central term mentioned by participants was “employer responsibility,” highlighting the role of employers in creating a work environment that promotes employees’ health, happiness, and productivity by fostering adequate working conditions and decent jobs. Participant 7 stated that sustainable employability involves the “ability to work and perform the tasks assigned to you up to the preferred retirement age. Creating ‘good’ jobs, with fair pay and good working conditions for individuals throughout their working lives. Work should be stable and secure, while also being personally rewarding”. In addition, other participants added: “Facilitating people’s productive work in the long term. This means promoting and protecting health, favorable working conditions, including contractual terms, and promoting work–life balance, including the reconciliation of work and care responsibilities (P6); “Sustainable work is the employer’s responsibility” (P3).

Finally, the responses also addressed the term “inclusion of older workers”, emphasizing the need to provide appropriate work for a diverse population and the importance of combating age-related prejudice in the labor market. As noted by participant 5: “…availability of work that is suitable for a diverse population, including older workers, that is not harmful to health and wellbeing and that is, in fact, safe”. The experts emphasized the importance of organizational responsibility for promoting diversity, fostering generational integration, and combating age discrimination.

*Axis 2—Organizational Practices.*

The analysis of Axis 2 revealed that organizational practices related to sustainable employability center on key concepts such as “flexibility”, “working conditions”, “development”, and “inclusion”. The analysis identified flexibility, healthy working conditions, continuous professional development, and the valorization of experience as the main organizational practices mentioned by the experts.

Flexibility emerged as a principal theme, encompassing the organization of work hours, location, and modalities. One participant emphasized that “one of the most effective practices is to be flexible in terms of working hours, including time, place, and type of work” (P14), further highlighting the critical role of autonomy in task execution. This perspective aligns with the notion of harmonizing job demands with individual capabilities, as reflected in the statement: “matching demands and skills and providing the resources employees need [...] will lead to sustaining people’s employability throughout life” (P2).

Another set of central terms refers to “healthy and safe working conditions”, including the promotion of mental health, work–life balance, and the provision of favorable psychosocial environments. Participants emphasize the importance of “promoting health at work” (P5) and ensuring “safe and healthy working conditions” (P11), reinforcing the role of organizations in risk prevention and in promoting wellbeing.

In addition, the analysis revealed the relevance of practices aimed at “continuous development” and “lifelong learning”, with an emphasis on training, upskilling, and reskilling workers. As participants pointed out, it is essential to “commit to providing training and developing older workers (P10)” and to ensure access to continuing education and opportunities for professional growth.

Another important aspect concerns the “valorization of experience” and the promotion of inclusive and intergenerational environments. Practices such as mentoring, recognition of accumulated knowledge, and combating ageism were highlighted, emphasizing the need to “strengthen spaces for value creation for people over 50 years old” (P13).

*Axis 3—Public Policies.*

In the public policy axis, the analysis revealed frequent associations between the terms “legislation”, “protection”, “incentives”, and “qualification”, indicating that sustainable employability is perceived as dependent on institutional structures that support continued participation in the labor market throughout life.

One of the main cores identified is combating age discrimination, as evidenced by recurring terms such as “anti-discrimination legislation” (P6) and “sanctions against arbitrary discrimination” (P4). Participants highlight the importance of policies that “combat age-related stereotypes” (P9) and promote equal opportunities in the labor market (P10).

Another central aspect involves the need for integration between different social protection systems, especially health, social security, and employment. In this sense, the relevance of “adequate and non-discriminatory pension policies” (P1) is emphasized, as well as actions that articulate different institutional spheres to ensure more stable work trajectories (P11).

Furthermore, terms such as “qualification”, “requalification”, and “lifelong learning” recur, underscoring the importance of policies for continuing education. Experts highlight the need for “hiring and training incentives” (P12) and “individual training vouchers for the entire working-age population” (P10), emphasizing the State’s role in promoting skills development.

Economic and regulatory mechanisms were also mentioned, such as tax incentives, flexibility in working conditions, and support for labor market reintegration (P1, P2, P9, P10). The experts highlighted legislation against age discrimination, the integration of health, social security and employment systems, qualification policies, and labor-market incentives as important public policy themes.

Considering this context, the items for the second round were designed to encompass different dimensions of public policy, including legislation against age discrimination, integration of protection systems, qualification and requalification programs, as well as hiring incentives and support for career transitions.

*Axis 4—Individual Factors.*

At the individual level, the analysis revealed the occurrence of central terms organized around the following categories: “health and wellbeing”, “flexibility and technological adaptation”, “education and continuous personal development”, and “social support networks”. The analysis identified four main categories of individual factors: health and wellbeing, flexibility and technological adaptation, education and continuous personal development, and social support networks.

The category “health and wellbeing” emerged as the primary semantic core, recognized as a fundamental prerequisite for maintaining employability. Physical and mental health were directly linked to work capacity, motivation, and adaptability to job demands. As participants emphasized, “health is the predominant factor” (P13), and it must be preserved throughout life to sustain employability, as reflected in the statement: “some aspects make a person sustainably employable if they are maintained stable throughout life, such as good health, a good level of competence, and wellbeing” (P1).

Another significant set concerns “flexibility and technological adaptation”, encompassing both the capacity to adjust working hours and arrangements and the readiness to meet emerging technological demands. These aspects underscore the necessity for continuous adaptation to labor market transformations, as illustrated by the statement: “flexibility in working hours; preparation to deal with new technological demands” (P2).

In addition, the analysis highlighted the importance of “education and continuous personal development”, emphasizing the centrality of lifelong learning and the constant updating of competencies (P12, P10). This factor is associated with adaptability, the construction of sustainable career trajectories, and the ability to cope with changes in the work context.

Finally, “social support networks” were identified as a crucial element, indicating that sustainable employability does not rely solely on isolated individual attributes but also on the social support accessible to workers (P12, P10, P9), including professional and personal relationships that facilitate continued labor market participation.

*Axis 5—Research Priorities.*

The analysis of research priorities highlighted the prominence of terms such as “health”, “ageism”, “interventions”, and “transformations of work”, signaling areas deemed strategic for advancing the field of sustainable employability. “Health and wellbeing at work” emerged as a principal priority, particularly amid an aging workforce. Participants stressed the importance of investigating “occupational health and wellbeing to address aging at work” (P8, P11), emphasizing the need to deepen understanding of the lifelong impacts of work on health.

Another salient theme concerned “ageism” and inequalities, encompassing issues related to gender, race, and precarious employment. As one participant noted, “minority ethnic groups are more likely to work in precarious positions” (P10), indicating the importance of intersectional approaches.

Moreover, the analysis highlighted the importance of studies on “transformations of work”, including digitalization, atypical work, and new forms of work organization (P3, P5, P6). These themes reflect concerns about structural changes that impact the employability of older workers.

Participants underscored the necessity for longitudinal studies and rigorous evaluation of interventions, emphasizing the critical importance of generating robust evidence on the long-term effectiveness of practices and policies (P1, P12). Additionally, emerging topics such as support networks and social inclusion were identified, albeit with less prominence, indicating promising avenues for future research.

### 3.2 Results of the second round (Delphi 2)

The qualitative analysis of the first round allowed for the identification of five central axes: (1) definition of sustainable employability, (2) organizational practices, (3) public policies, (4) individual factors, and (5) research priorities. These axes encompassed the main elements identified by the experts regarding the sustainability of employability among older workers and served as the basis for the construction of the second-round instrument. The progression from the qualitative themes identified in the first Delphi round to the questionnaire items developed for the second round is presented in Supplementary Table S2.

Building upon the themes identified within each analytical domain, the qualitative findings were operationalized into objective statements addressing multiple dimensions, including the maintenance of work capacity throughout life, organizational responsibility, inclusion of older workers, specific organizational practices (such as work flexibility, promotion of healthy environments, professional development, valorization of experience, and combating ageism), individual factors (such as health, motivation, self-efficacy, adaptability, and lifelong learning), public policies, and priorities for future research agendas. These statements formed the quantitative questionnaire administered in the second round, in which participants were invited to rate their level of agreement with them.

The second round aimed to assess the level of agreement among experts regarding the items derived from the qualitative analysis of the first round. Participants evaluated each statement on a Likert scale, enabling analysis of consensus levels using descriptive statistics.

Overall, the results indicated high levels of agreement for most items, with a predominance of responses at the upper levels of the scale and low variability among experts. Most items received high levels of agreement, with responses concentrated on the upper categories of the scale and low variability among experts.

In this round, the definition of sustainable employability was examined using statements that directly reflected the central elements identified in the initial qualitative analysis. Specifically, three statements were formulated to operationalize the key emerging semantic cores: (1) sustainable employability constitutes an individual’s continuous capacity to remain active and fulfilled in the labor market, adapting to changes while securing stable and rewarding employment; (2) employers hold a pivotal role in fostering sustainable employability by providing favorable working conditions, promoting health, and ensuring meaningful work; and (3) sustainable employability encompasses equitable access to opportunities for diverse workers, particularly older adults, necessitating the eradication of age-based discrimination.

The three statements representing the definition of sustainable employability all achieved the predefined consensus criteria (Table 2). Experts consistently endorsed sustainable employability as a continuous capacity to remain in work throughout life, emphasized the employer’s responsibility for creating favorable working conditions, and highlighted the importance of inclusion and the prevention of age discrimination.

**Table 2:** Degree of agreement among experts on the Delphi2 items.

Domain	Item	Md	IQR	% Agreement	Consensus
Definition of sustainable employability	Sustainable employability constitutes an individual’s continuous capacity to remain active and fulfilled in the labor market, adapting to changes while securing stable and rewarding employment	5,0	1,0	75,0	Achieved
	Employers hold a pivotal role in fostering sustainable employability by providing favorable working conditions, promoting health, and ensuring meaningful work	5,0	0,5	91,7	Achieved
	Sustainable employability encompasses equitable access to opportunities for diverse workers, particularly older adults, necessitating the eradication of age-based discrimination,	5,0	0,5	91,7	Achieved
Individual factors	Physical and mental health	5.0	1.0	83.3	Achieved
	Motivation and engagement at work	5.0	1.0	91.7	Achieved
	Social–emotional skills	5.0	1.0	91.7	Achieved
	Lifelong learning	5.0	1.0	91.7	Achieved
	Flexible schedules	4.5	1.0	91.7	Achieved
	Satisfaction with coworkers	4.5	1.0	91.7	Achieved
	Formal education	4.0	1.5	58.3	Not achieved
Organizational practices	Healthy and safe work environments	5.0	0.0	91.7	Achieved
	Combating ageism	5.0	0.5	91.7	Achieved
	Mental health and wellbeing promotion	5.0	0.5	91.7	Achieved
	Valuing experience	5.0	1.0	91.7	Achieved
	Flexibility at work	5.0	1.0	91.7	Achieved
	Professional development programs	5.0	1.0	91.7	Achieved
Public policies	Legislation against age discrimination	5.0	1.0	91.7	Achieved
	Integration of health care, social security, and employment	5.0	1.0	91.7	Achieved
	Training and retraining programs	4.0	0.5	91.7	Achieved
	Promoting lifelong learning	4.0	1.0	91.7	Achieved
	More protective contracts	4.0	2.5	58.3	Not achieved
	Tax incentives for companies	4.0	1.0	58.3	Not achieved
Research priorities	Health and wellbeing at work	5.0	1.0	91.7	Achieved
	Inequalities among minority groups	5.0	1.0	91.7	Achieved
	Precarious employment	5.0	1.0	91.7	Achieved
	Support networks	3.0	1.0	41.7	Not achieved

Md = median; IQR = interquartile range; % Agreement = percentage of experts who assigned responses in the upper categories of the Likert scale. The results refer to the second round of the Delphi study (*n* = 12).

In the domain of individual factors, strong agreement was observed regarding the importance of physical and mental health, motivation and work engagement, as well as socioemotional skills. Lifelong learning also showed high agreement, reinforcing its importance for maintaining employability. Aspects related to the relational and organizational context, such as satisfaction with coworkers and flexibility in working hours, were also highly valued. In contrast, the item referring to formal education showed a lower level of agreement.

Concerning organizational practices, the results showed high consensus for the promotion of healthy and safe work environments, as well as for combating ageism and promoting mental health and wellbeing. The valorization of older workers’ experience and the provision of work flexibility were also recognized as relevant practices. In addition, continuous professional development programs showed a high level of agreement, reinforcing the importance of organizational investment in skills updating.

In the field of public policies, strong convergence was observed regarding the need for robust legislation against age discrimination and the integration of health, social security, and employment policies. Professional qualification and requalification programs and the promotion of lifelong learning were also supported. In contrast, items related to more specific mechanisms, such as employment contracts that make it more difficult to dismiss older workers and tax incentives for companies, showed greater dispersion in responses, indicating lower consensus among experts.

Finally, in the domain of research priorities, a high level of agreement was observed for health and wellbeing at work, inequalities among minority groups, and precarious employment. In contrast, the item related to support networks showed lower agreement, suggesting divergent views on its centrality in the field of sustainable employability. Table 2 presents the complete set of items evaluated in the second round, along with their respective agreement indicators.

Therefore, the results of the second round indicate consistent consensus among experts on the main factors that sustain employability throughout life, particularly individual resources and organizational practices. On the other hand, the greater variability observed in items related to specific public policies and support networks suggests that these aspects remain open and depend on contextual factors, which justified their reintroduction in the third round of the study.

Based on the predefined consensus criteria (Md ≥ 4, IQR ≤ 1.0, and ≥75% agreement), consensus was achieved for 22 of the 26 items evaluated in the second Delphi round. The remaining four items did not meet one or more of the predefined criteria and were therefore retained for reassessment in the third Delphi round.

### 3.3 Results of the third round (Delphi 3)

The study’s third round aimed to deepen the analysis of items that had not reached consensus in the previous stage and to evaluate new items suggested by the experts based on the qualitative responses from the second round. Nine experts participated in this stage, reassessing the reintroduced items and judging the relevance of the new elements incorporated into the instrument.

In this round, a trend toward increased agreement was observed for some of the reintroduced items, particularly those related to organizational and contextual aspects of sustainable employability. However, some re-evaluated and newly proposed policy-related items continued to show lower or only moderate agreement, particularly tax incentives for companies, funding for age-friendly adaptations, and, to a lesser extent, contracts that make it difficult to terminate the employment of older workers. These findings suggest persistent divergences regarding institutional mechanisms intended to support longer and more sustainable working lives (Table 3).

**Table 3:** Results of the third round (Delphi3): resubmitted items and new items.

Item type	Domain	Item	Md	IQR	% Agreement	Consensus
Revalued	Individual factors	Formal education	5.0	2.0	66.7	Not achieved
Revalued	Public policy	Contracts that make it difficult to terminate the employment of older workers	4.0	1.0	77.8	Achieved
Revalued	Public policy	Tax incentives for companies	4.0	1.0	55.6	Not achieved
Revalued	Research	Support networks	4.0	1.0	77.8	Achieved
New	Individual factors	Meaning or purpose at work	5.0	1.0	88.9	Achieved^*^
New	Individual factors	Career adaptability	4.0	1.0	88.9	Achieved^*^
New	Individual factors	Autonomy and self-regulation	4.0	2.0	66.7	Not achieved^*^
New	Individual factors	Work–life balance strategies	4.0	1.0	77.8	Achieved^*^
New	Public policy	Funding for age-friendly adaptations	4.0	1.0	66.7	Not achieved^*^
New	Public policy	Recognition of prior learning	5.0	1.0	77.8	Achieved^*^
New	Public policy	Anti-ageism campaigns	5.0	1.0	77.8	Achieved^*^
New	Public policy	Policy-regulated flexible arrangements	5.0	1.0	100.0	Achieved^*^
New	Public policy	Career transition support	5.0	1.0	77.8	Achieved^*^

Md = median; IQR = interquartile range; % Agreement = percentage of experts who assigned responses in the upper categories of the Likert scale. Results refer to the third round of the Delphi study (*n* = 9), consisting of items reintroduced from the previous round and new items derived from participants’ suggestions. ^*^These items met the predefined consensus criteria (Md ≥ 4, IQR ≤ 1.0, and ≥75% agreement) but were introduced only in Round 3 and therefore underwent a single quantitative assessment rather than the full iterative Delphi consensus process. Consequently, their level of evidence should be interpreted separately from that of the re-evaluated items.

Furthermore, the inclusion of new items broadened understanding of the construct by incorporating dimensions related to career trajectories, the meaning of work, and the institutional regulation of working conditions. These new items were not inserted arbitrarily but emerged from the qualitative suggestions provided by the experts at the end of the second round, in a dedicated space for comments, justifications, and proposals for aspects not yet covered in the instrument. This procedure enabled the incorporation of elements considered relevant by the panel, such as ergonomic issues and other contextual factors related to the sustainability of employability among older workers, thereby reinforcing the iterative and participatory nature of the Delphi technique. Table 3 presents the detailed results of the third round, including both the reintroduced items and the new items incorporated into the instrument.

Overall, the Delphi process resulted in consensus on the definition of sustainable employability, multiple individual factors, organizational practices, and public policy dimensions. Consensus was consistently achieved for health-related factors, lifelong learning, healthy work environments, organizational practices addressing ageism, workplace flexibility, and the integration of health, social security, and employment policies. Additional dimensions, including meaning of work, career adaptability, work–life balance, and support networks, achieved consensus in the third round. In contrast, tax incentives for companies and funding for age-friendly workplace adaptations did not reach the predefined consensus criteria by the end of the Delphi process.

## 4 Discussion

The study’s results were analyzed by integrating the qualitative and quantitative stages, thereby advancing the understanding of the employability of older workers in an empirically grounded manner. Considering the study’s objective of identifying factors, practices, policies, and dimensions that achieved expert consensus.

The results showed that the conceptual structure of sustainable employability identified in the first Delphi round remained largely consistent across the subsequent consensus rounds. The qualitative analysis enabled the identification of recurrent themes within the five analytical domains explored in the structured elicitation. The high levels of agreement observed for most of these items indicate that these dimensions achieved strong expert consensus within the participating panel.

This result is relevant considering recurring critiques in the literature on sustainable employability, which point to conceptual fragmentation and a lack of integration between levels of analysis (De Lange et al., 2022; Fleuren et al., 2020). In this study, the convergence between stages suggests that, at least at the level of expert consensus, there is a structured core that articulates individual, organizational, and governmental factors. This empirical articulation supports the conception of sustainable employability as a multilevel construct, in line with more recent proposals (Nilsson et al., 2023; Le Blanc et al., 2017).

Regarding the definition of the construct, the results the existence of a core set of dimensions that consistently achieved expert consensus, composed of three central elements: the continuous capacity to remain in work throughout life, the influence of working conditions in sustaining this continuity, and the need for inclusion. This empirical delimitation helps reduce the conceptual ambiguity still present in the field (Fleuren et al., 2016) by making explicit the components that constitute sustainable employability. At the same time, the incorporation of dimensions such as meaning of work, career adaptability, and work–life balance broadens this definition, bringing it closer to discussions on sustainable careers, in which continuity, meaning, and wellbeing are considered central dimensions of professional trajectories (De Vos et al., 2020; Donald et al., 2024).

At the individual level, there was high agreement regarding variables such as physical and mental health, motivation, socioemotional skills, lifelong learning, flexible work schedules and satisfaction with coworkers. In contrast, formal education showed lower consensus among experts. One possible explanation for this finding is the ongoing transformation of the world of work, in which maintaining employability increasingly depends on workers’ capacity for adaptation, continuous learning, and the mobilization of accumulated experience rather than on formal educational credentials alone. This interpretation is consistent with the AMO model (Ability–Motivation–Opportunity), which proposes that sustainable work participation depends on the interaction between workers’ abilities, motivation, and opportunities provided by organizations (Le Blanc et al., 2017; Gürbüz et al., 2023).

Some themes identified in the individual domain, particularly flexible work schedules and satisfaction with coworkers, may also be interpreted as organizational or contextual characteristics. However, these items were retained within the domain in which they were originally identified during the qualitative stage to preserve the structure of the experts’ responses and maintain consistency between the qualitative and quantitative phases of the Delphi process. This overlap reflects the inherently multilevel nature of sustainable employability, in which certain dimensions cannot be exclusively assigned to a single level of analysis.

The limitation of an approach focused exclusively on the individual became even more evident when examining the results regarding organizational practices. The high level of consensus regarding factors such as healthy work environments, flexibility, and valorization by organizational structures. Consequently, the data support the idea that opportunity, in the AMO model, should be treated as a structuring dimension of the construct (Floris and Pinna, 2024; Gürbüz et al., 2024).

These results are supported by the Swage model (Nilsson, 2020), which posits that continued participation in work stems from interactions among health, the work environment, competencies, and motivation throughout the life course. The findings also converge with this perspective by showing that factors such as health and work do not operate independently, but rather as interdependent dimensions that influence the ability to continue working (Deng et al., 2021). The strong agreement observed for items related to health and organizational conditions suggests that sustainable employability is not a static attribute, but rather a dynamic balance between demands and resources (Stuer et al., 2019).

The international composition of the panel constitutes both a strength and a challenge. By bringing together experts from countries with different labor market structures, pension systems, welfare regimes, and employment regulations, the study broadens the range of perspectives considered in defining sustainable employability. At the same time, the diversity of institutional contexts represented in the panel may have contributed to the comparatively lower consensus observed for some policy-related items. However, this interpretation should be viewed with caution, as panel attrition reduced the representation of some countries in the final Delphi round. Because these institutional arrangements differ substantially across countries, their perceived relevance and feasibility are likely to vary according to specific socioeconomic, legislative, and labor market contexts (Van Dalen et al., 2019; Vickerstaff and Van der Horst, 2021).

These findings are consistent with previous studies suggesting that governmental dimensions of sustainable employability are inherently context-dependent and therefore more difficult to conceptualize uniformly than individual or organizational dimensions (Van der Klink et al., 2016; De Lange et al., 2022). Rather than indicating that policy-related factors are less important, the lower level of consensus suggests that their implementation and perceived effectiveness depend on broader institutional conditions, including social protection systems, employment legislation, and retirement policies. Consequently, these findings reinforce the importance of adopting multilevel approaches that explicitly consider the interaction between individual, organizational, and policy-related factors when studying sustainable employability, while highlighting the need for greater theoretical refinement and empirical investigation of policy-related dimensions (Fleuren et al., 2020; Mo et al., 2024). Future comparative studies involving experts from different welfare-state regimes may help distinguish policy priorities that are broadly applicable from those that are context-specific.

The third round reinforces this interpretation by showing that, even after the iterative process characteristic of the Delphi method, certain items maintain moderate levels of agreement. However, the inclusion of new elements, such as meaning of work, career adaptability, and work–life balance, broadens the understanding of the construct by incorporating more processual and subjective dimensions. These findings should be interpreted as promising dimensions identified by the expert panel rather than as fully established Delphi consensus, since these items were introduced and evaluated during only one quantitative round. These results are aligned with the literature on sustainable careers, which emphasizes continuity, meaning, and wellbeing as central dimensions of professional trajectories over the life course (De Vos et al., 2020; Donald et al., 2024). Furthermore, the relevance attributed to career adaptability is consistent with perspectives that highlight the ability to cope with transitions and changes as a fundamental element for the sustainability of career trajectories (Savickas, 2013; Rudolph et al., 2017), while the centrality of meaning of work reinforces evidence that meaningful work experiences are directly associated with job retention and wellbeing (Allan et al., 2019). However, organizations in general must be aware of the measures required to address the needs of older workers in the context of inclusion (França et al., 2017). Taken together, these elements indicate a shift in sustainable employability from a strictly functional perspective to an approach that incorporates subjective and relational dimensions.

From a theoretical standpoint, these results allow for advancement in two directions. First, by empirically demonstrating the articulation between different levels of analysis, the study helps reduce conceptual fragmentation in the field. Second, by showing that the level of expert agreement varied across dimensions, the findings suggest greater convergence regarding individual and organizational factors than policy-related dimensions. These differences should be interpreted as reflecting the opinions of the participating expert panel rather than definitive evidence of differences in the stability of the construct itself. This distinction is relevant for guiding both theoretical development and the formulation of policies and practices, indicating where there is greater conceptual clarity and where interpretative disputes persist, also considering the socioeconomic characteristics of the population, changes in pension systems and their sustainability, and existing inequalities. This may help explain why, in some countries where older workers most desire (and/or need) to continue working, sustainable employability becomes more necessary.

Regarding research priorities, the results provide consistent indications for advancing the field. The consensus on health and wellbeing at work, inequalities among minority groups, and precarious employment highlights the importance of including these dimensions in future research agendas, aligning with findings from other studies (Neupane et al., 2022; Mo et al., 2024). In addition, the lower level of consensus regarding support networks suggests theoretical and empirical gaps that require further investigation into the delimitation and operationalization of this dimension of sustainable employability. Furthermore, the lower importance attributed to formal education, in contrast with the value placed on lifelong learning, indicates the need to investigate less linear and more adaptive career trajectories. This result is consistent with contemporary transformations in work, marked by greater instability, technological changes, and continuous demands for skill updating (Cascio and Montealegre, 2016; Van Dam et al., 2017), as well as with perspectives that emphasize continuous learning as a central element for the sustainability of careers throughout life (Le et al., 2023; De Vos et al., 2020).

From a practical standpoint, the results indicate that promoting sustainable employability requires interventions that integrate different levels. At the organizational level, the need for investments in working conditions that support health, flexibility, and inclusion was highlighted. At the level of public policies, the results suggest the importance of broad guidelines, such as combating discrimination and integrating systems. These implications are particularly relevant in the context of population aging and the need to extend working lives (Fisher et al., 2016). These findings suggest that organizations should prioritize interventions aimed at promoting healthy working environments, combating age discrimination, expanding opportunities for lifelong learning, and offering flexible work arrangements. Likewise, policymakers should strengthen integrated employment, health, and retirement policies capable of supporting longer and healthier working lives.

Finally, the results point to the need to advance in the integration of objective and subjective dimensions of sustainable employability. Although the study contributes to the conceptual clarification of sustainable employability among older workers through expert consensus, important theoretical challenges remain regarding the articulation of continued participation in work, working conditions, meaning of work, and wellbeing throughout the life course. In this sense, future research may benefit from longitudinal and comparative designs, as well as from the development of measurement instruments that capture the dynamic, multilevel, and contextual nature of sustainable employability.

Although the present study provides an expert-informed framework for understanding sustainable employability among older workers, several limitations should be considered when interpreting the findings. First, the Delphi panel consisted exclusively of academic experts. Although this choice was appropriate for obtaining expert consensus regarding conceptualization, organizational practices, public policies, and research priorities associated with sustainable employability, the findings primarily reflect an academic perspective and may not fully capture the views of other relevant stakeholders, such as employers, policymakers, and older workers themselves. Future studies should integrate these perspectives to provide a more comprehensive understanding of sustainable employability. Notably, the present study constitutes one stage of a broader research program that will also investigate these stakeholder groups.

Second, recruiting and retaining internationally recognized experts throughout the Delphi process represented an important methodological challenge. Although panel attrition is common in Delphi studies and the final number of participants remained within the range considered appropriate for expert panels, the reduction from 14 experts in the first round to 9 in the final round may have increased the influence of individual judgments on the consensus indicators. The panel also experienced attrition across rounds, decreasing from 14 experts in Round 1 to 12 in Round 2 and nine in Round 3. Although the final panel retained geographical diversity, with experts from seven countries across Latin America, Europe, and Oceania, the distribution across countries was uneven, with Brazil represented by two experts and the other countries by one expert each. This composition should be considered when interpreting findings related to institutional and welfare-regime contexts.

Also, because the final Delphi round included nine participants, the percentage agreement criterion was relatively sensitive to individual ratings, meaning that a single response could determine whether the predefined 75% agreement threshold was reached. Although the consensus criteria were established *a priori* and combined median, interquartile range, and percentage agreement to reduce reliance on a single indicator, future Delphi studies with larger expert panels may provide more stable estimates of consensus.

In addition, several themes incorporated into the third Delphi round achieved the predefined consensus criteria despite being evaluated only once. Accordingly, these findings should be interpreted as promising dimensions identified by the expert panel rather than as dimensions that completed the full iterative Delphi consensus process. Future studies should reassess these themes in additional Delphi rounds to strengthen the stability of the observed consensus.

Finally, as with Delphi studies in general, the findings reflect expert consensus regarding sustainable employability rather than direct observations of how employability evolves over time. Consequently, longitudinal studies following older workers across different career stages are needed to examine the dynamic interactions among individual, organizational, and contextual factors throughout working life. Future research should also broaden participant diversity by including different occupational groups, employment sectors, and national contexts, thereby contributing to a more comprehensive understanding of sustainable employability across diverse settings.

## 5 Final considerations

This study contributed to the conceptual clarification of sustainable employability among older workers by integrating expert perspectives through the Delphi technique. The findings identified a set of dimensions that consistently achieved expert consensus, encompassing individual factors, organizational practices, governmental policies, and research priorities relevant to sustainable employability. Rather than proposing a definitive conceptual model, the study offers an expert-informed framework that may support future theoretical refinement, instrument development, organizational interventions, and public policy initiatives.

Beyond contributing to conceptual clarification, the study provides a structured set of priorities that may inform future research agendas and support evidence-based decision-making in organizations and public policies. By identifying areas of stronger and weaker expert consensus, the findings also highlight domains requiring further theoretical development and empirical investigation, particularly those related to governmental actions and institutional contexts.

Future research should extend these findings by incorporating the perspectives of employers, policymakers, and older workers, as well as by conducting longitudinal and cross-cultural studies capable of examining how sustainable employability develops over time across different institutional contexts. In addition, future psychometric studies may build on the dimensions identified in this Delphi process to develop and validate measurement instruments for sustainable employability among older workers. Taken together, these findings contribute to advancing sustainable employability among older workers from a concept characterized by considerable theoretical fragmentation toward a more structured, expert-informed framework capable of guiding future research, organizational practice, and public policy.

## Data Availability

The raw data supporting the conclusions of this article will be made available by the authors, without undue reservation.
