# Supplementary material for: Expert consensus on sustainable employability of older workers: research, practice, and policy dimensions
Source: Front Psychol. 2026 Sep 14;17:1903399. doi: 10.3389/fpsyg.2026.1903399 (PMC13616651; doi:10.3389/fpsyg.2026.1903399)
Supplement: Supplementary file 1 [file Table_1.DOCX]

**Sustainable Employability of Workers Aged 45+**

*Delphi Study — Questionnaires, Traceability, and Controlled Feedback*

Supplementary Materials S1–S5

**SUPPLEMENTARY MATERIAL**

**Supplementary Material S1. Delphi Round 1 questionnaire.**

**The First Round of the Delphi Study (Delphi1) – Sustainable Employability of Workers Aged 45+**

**Instructions for Participation in the Delphi Study**

**About the Questionnaire:**

- This study involves three rounds of questionnaires, each round building upon responses from the previous one. Your input will be crucial to help identify areas of convergence and divergence among experts throughout these rounds.
- In this first round, you will find open-ended and multiple-choice questions, where you can share your detailed perspectives and suggestions on specific aspects of the Delphi method.
- Based on the responses collected, subsequent rounds will be more targeted and will build upon the themes and items generated from the contributions provided in this round, with quantitative assessment introduced in the following stage.
- **Instructions for Answering:**
- Respond openly and according to your professional experience.
- Carefully read each question and provide complete and detailed answers whenever possible.
- The estimated time to complete this first round is approximately 10 minutes.

Response Deadline: Please complete this round within 15 days of receiving this invitation.

We sincerely appreciate your collaboration and the contribution of your expertise to this collective knowledge-building process. If you have any questions, feel free to contact us at luizasccontage@gmail.com

Thank you for your participation and commitment.

# Part 1: Open questions

Please read each question carefully and answer it objectively. If you think it necessary, you can add comments and suggestions to each item.

1. What does sustainable employability mean to you? (between 30- 50 words)
2. In your opinion, what are the most effective organizational practices for promoting the sustainable employability of workers aged 45+?  (between 30- 50 words)
3. What public policies do you believe are the key to support the sustainable employability of 45+ workers?  (between 20- 50 words)
4. What individual factors contribute to the sustainable employability of workers aged 45+?  (between 20- 50 words)
5. What do you think are the priority topics for research into the sustainable employability of workers aged 45+?  (between 20- 50 words)

# Part 2: Sociodemographic Questionnaire

Please provide some information about your professional and academic profile. This will allow us to better contextualize your answers:

1. Age: __________________________________________________________
2. Gender:

( ) Male

( ) Female

( ) Other: __________________

3. Country: _________________________________________________________
4. Subject area/ Current position: _______________________________________
5. How long have you been working in the field of employability or related area:

( ) Less than 5 years

( ) 5 to 10 years

( ) 11 to 20 years

( ) More than 20 years

6. In the next round of this survey**,** do you agree to continue participating in this survey?

( ) Yes

( ) No

**Thank you for your participation!**

After the responses from this first round have been analyzed, **you will receive a new questionnaire after a month for the second phase of the study**.

**Supplementary Table S2. Traceability of qualitative findings from Delphi Round 1 and corresponding questionnaire items developed for Delphi Round 2**

| **Delphi Round 1 Axis** | **Main themes / subthemes identified** | **Representative quotation(s)** | **Corresponding Delphi Round 2 item(s)** |
| --- | --- | --- | --- |
| **1. Definition of sustainable employability** | • Continuous ability to remain employed throughout life • Adaptation to changes • Good working conditions • Employer responsibility • Inclusion and age-related bias | “Sustainable employability is enabling individuals to be employed for as long and as continuously as possible…” (P3). “It implies the possibility of continuing to be employable throughout life, especially in relation to possible age-related biases.” (P4). “Sustainable work is the employer’s responsibility.” (P3). | • Continuous capacity to remain employed throughout life • Working conditions that sustain employability • Inclusion of older workers |
| **2. Individual factors** | • Physical and mental health • Motivation • Social-emotional competencies • Lifelong learning • Flexibility and adaptation • Satisfaction with coworkers • Formal education | “Health is the predominant factor.” (P13). “Some aspects make a person sustainably employable… good health…” (P1). “Flexibility in working hours; preparation to deal with new technological demands.” (P2). | • Physical and mental health • Motivation and engagement at work • Social-emotional skills • Lifelong learning • Flexible schedules • Satisfaction with coworkers • Formal education |
| **3. Organizational practices** | • Healthy and safe work environments • Flexibility at work • Mental health and well-being • Professional development • Valuing experience • Combating ageism | “Creating ‘good’ jobs, with fair pay and good working conditions…” (P7). “Facilitating people’s productive work in the long term…” (P6). | • Healthy and safe work environments • Combating ageism • Mental health and well-being promotion • Valuing experience • Flexibility at work • Professional development programs |
| **4. Public policies** | • Legislation against age discrimination • Integration of health, employment and social security • Training and retraining • Lifelong learning • Protective employment contracts • Tax incentives | “Adequate and non-discriminatory pension policies…” (P1). “Hiring and training incentives…” (P12). “Individual training vouchers…” (P10). | • Legislation against age discrimination • Integration of health care, social security, and employment • Training and retraining programs • Promoting lifelong learning • More protective contracts • Tax incentives for companies |
| **5. Research priorities** | • Health and well-being • Ageism • Minority inequalities • Precarious employment • Transformations of work • Longitudinal studies • Evaluation of interventions • Support networks | “Occupational health and well-being to address aging at work.” (P8, P11). “Minority ethnic groups are more likely to work in precarious positions.” (P10). | • Health and well-being at work • Inequalities among minority groups • Precarious employment • Support networks |

**Note.** The thematic categories emerged from the qualitative analysis of the first Delphi round using the Requalify.ai platform and were subsequently reviewed and interpreted by the first and last authors. These categories served as the basis for generating the questionnaire items evaluated in the second Delphi round.

**Supplementary Material S3. Delphi Round 2 questionnaire.**

**Second Round of the Delphi Study (Delphi2) – Sustainable Employability of Workers Aged 45+**

**Instructions for Participation in the Delphi Study – Second Round**

**About the Questionnaire:
This is the second round of a three-round Delphi study. The responses from the first round composed of open-ended were carefully analyzed and synthesized. Based on your valuable qualitative input, we have developed this second-round questionnaire using Likert-type scale items.**

Your participation is essential to refine and validate the key themes, practices, policies, and research areas identified so far. The goal of this round is to assess the level of agreement among experts and move toward greater consensus.

**Instructions for Answering:**

- For each statement, please indicate your level of agreement using the Likert scale provided.
- In addition to selecting a response, we kindly ask that you provide brief comments or justifications for your answers, especially when you disagree or strongly disagree, or if you have suggestions for improvement or clarification.
- Your feedback will be instrumental in shaping the final round of this study.

**Estimated Time:** Approximately 15–20 minutes.

**Response Deadline:** Please complete this round within 20 days of receiving this invitation.

We sincerely appreciate your continued collaboration and the valuable contribution of your expertise to this collective process of knowledge building. If you have any questions or difficulties accessing the questionnaire, feel free to contact us at luizasccontage@gmail.com.

Thank you again for your participation and commitment.

# Section 1: Meaning of Sustainable Employability

Please read each statement carefully and indicate your level of agreement with the definitions and core aspects of what constitute sustainable employability. Use the scale below. When possible, add a comment to justify your response, especially if you disagree or strongly disagree, or if you have suggestions for improvement.

| **Item** | **Strongly disagree** | **Disagree** | **Neutral** | **Agree** | **Strongly agree** |
| --- | --- | --- | --- | --- | --- |
| Sustainable employability is an individual's ongoing ability to remain active and satisfied in the labor market, adapting to change and securing work that is secure and rewarding. |  |  |  |  |  |
| Employers play a central role in enabling sustainable employability by providing good working conditions, promoting health, and offering meaningful work. |  |  |  |  |  |
| Sustainable employability includes fair access to opportunities for diverse workers, especially older individuals, and requires combating age-based discrimination. |  |  |  |  |  |

 Do you have any comments or suggestions about this section?

____________________________________________________________________________________________________________________________________________

# Section 2: Individual Factors

Please indicate your level of agreement with the individual elements that support sustainable employability.

| **Item** | **Strongly disagree** | **Disagree** | **Neutral** | **Agree** | **Strongly agree** |
| --- | --- | --- | --- | --- | --- |
| Physical and mental health |  |  |  |  |  |
| Motivation and engagement at work |  |  |  |  |  |
| Social-emotional skills |  |  |  |  |  |
| Lifelong learning |  |  |  |  |  |
| Flexible schedules |  |  |  |  |  |
| Satisfaction with coworkers |  |  |  |  |  |
| Formal education |  |  |  |  |  |

  Comment:

____________________________________________________________________________________________________________________________________________

# Section 3: Organizational Practices

Please indicate your level of agreement with the organizational practices that support sustainable employability.

| **Item** | **Strongly disagree** | **Disagree** | **Neutral** | **Agree** | **Strongly agree** |
| --- | --- | --- | --- | --- | --- |
| Healthy and safe work environments |  |  |  |  |  |
| Combating ageism |  |  |  |  |  |
| Mental health and well-being promotion |  |  |  |  |  |
| Valuing experience |  |  |  |  |  |
| Flexibility at work |  |  |  |  |  |
| Professional development programs |  |  |  |  |  |

  Comment:

____________________________________________________________________________________________________________________________________________

# Section 4: Public Policies

Please indicate your level of agreement with the public policies that support sustainable employability.

| **Item** | **Strongly disagree** | **Disagree** | **Neutral** | **Agree** | **Strongly agree** |
| --- | --- | --- | --- | --- | --- |
| Legislation against age discrimination |  |  |  |  |  |
| Integration of health care, social security, and employment |  |  |  |  |  |
| Training and retraining programs |  |  |  |  |  |
| Promoting lifelong learning |  |  |  |  |  |
| More protective contracts |  |  |  |  |  |
| Tax incentives for companies |  |  |  |  |  |

Comment:

____________________________________________________________________________________________________________________________________________

# Section 5: Research Priorities

Please indicate your level of agreement with the proposed research priorities related to sustainable employability.

| **Item** | **Strongly disagree** | **Disagree** | **Neutral** | **Agree** | **Strongly agree** |
| --- | --- | --- | --- | --- | --- |
| Health and well-being at work |  |  |  |  |  |
| Inequalities among minority groups |  |  |  |  |  |
| Precarious employment |  |  |  |  |  |
| Support networks |  |  |  |  |  |

Comment:

____________________________________________________________________________________________________________________________________________

**Thank you for your participation!**

**Supplementary Material S4. Delphi Round 3 questionnaire.**

**Final Round of Delphi Study (Delphi 3) - Sustainable Employability of Workers Aged 45+**

**Instructions for Participation in the Delphi Study – Third Round**

We would like to begin by expressing our sincere gratitude for your continued participation and thoughtful contributions throughout this study. Your engagement in the previous rounds has been invaluable in shaping and advancing this research.

This is the third and final round of a Delphi study conducted as part of a doctoral research project in Psychology. The responses from the previous round were carefully analyzed. Based on your valuable feedback, this final questionnaire includes:

- New items suggested by participants in the previous round;

# Items that did not reach sufficient consensus in the last round, now reformulated when necessary.

Your participation in this final step is essential to consolidate the findings, refine the proposed items, and achieve a stronger consensus among experts.

**Instructions for Answering**

- For each statement, please indicate your level of agreement using the Likert scale provided (1 = Strongly disagree to 5 = Strongly agree).
- In addition to selecting a response, we kindly ask that you provide brief comments or justifications, particularly for items that did not reach consensus previously, as your perspective will be crucial to understanding points of divergence.
- You may also include suggestions for clarification or improvement whenever you deem it appropriate.

# Items that did not achieve consensus in the previous round

| **Item** | **Strongly disagree** | **Disagree** | **Neutral** | **Agree** | **Strongly agree** |
| --- | --- | --- | --- | --- | --- |
| **Individual Factors:** |  |  |  |  |  |
| Formal education |  |  |  |  |  |
| **Public Policy:** |  |  |  |  |  |
| More protective contracts |  |  |  |  |  |
| Tax incentives for companies |  |  |  |  |  |
| **Research Priority:** |  |  |  |  |  |
| Support networks |  |  |  |  |  |

Comment:

____________________________________________________________________________________________________________________________________________

# Items proposed by participants in the previous round

**Please read each statement carefully and indicate your level of agreement with the definitions and core aspects of what constitute sustainable employability. Use the scale below. When possible, add a comment to justify your response, especially if you disagree or strongly disagree, or if you have suggestions for improvement.**

| **Item** | **Strongly disagree** | **Disagree** | **Neutral** | **Agree** | **Strongly agree** |
| --- | --- | --- | --- | --- | --- |
| **Individual Factors:** |  |  |  |  |  |
| Meaning of purpose at work |  |  |  |  |  |
| Career adaptability |  |  |  |  |  |
| Autonomy and self-regulation |  |  |  |  |  |
| Work-life balance strategies |  |  |  |  |  |
| **Public Policy:** |  |  |  |  |  |
| Funding for age-friendly adaptations |  |  |  |  |  |
| Recognition of prior learning |  |  |  |  |  |
| Anti-ageism campaigns |  |  |  |  |  |
| Policy-regulated flexible arrangements |  |  |  |  |  |
| Career transition support |  |  |  |  |  |

Comment:

____________________________________________________________________________________________________________________________________________

**Supplementary Material S5. Controlled Feedback**

**CONTROLLED FEEDBACK**

**Delphi Round 2 → Delphi Round 3**

*Sustainable Employability of Older Workers (45+)*

# 1. Introduction

**Purpose.** This form provided controlled quantitative feedback from Delphi Round 2 before the third Delphi round. Participants received the consolidated results for the items that did not reach the predefined consensus criteria and were invited to reassess those items and evaluate new items suggested during Round 2.

| **Indicator** | **Criterion** | **Purpose** |
| --- | --- | --- |
| **Median (Md)** | Md ≥ 4 | Central tendency |
| **Interquartile range (IQR)** | IQR ≤ 1.0 | Response dispersion |
| **Agreement** | ≥ 75% selecting 4 or 5 | Level of agreement |

**Feedback components.** For each item, the feedback included the group median, interquartile range, percentage of agreement, consensus status, and anonymized expert comments.

# 2. Summary of Delphi Round 2 Results

**The following four items did not meet one or more of the predefined consensus criteria and were therefore reintroduced for reassessment in Delphi Round 3.**

| **Domain** | **Item** | **Md** | **IQR** | **% Agreement** | **Consensus** |
| --- | --- | --- | --- | --- | --- |
| Individual factors | **Formal education** | 4.0 | 1.5 | 58.3% | **Not achieved** |
| Anonymized comments: “Formal education is relevant but not decisive for sustainable employability later in life.” “Experience and continuous learning matter more than initial education.” “Depends heavily on the occupational sector.” | | | | | |
| Public policies | **More protective contracts** | 4.0 | 2.5 | 58.3% | **Not achieved** |
| Anonymized comments: “May unintentionally reinforce stereotypes or reduce hiring incentives.” “Protection is important, but rigid contracts can backfire.” | | | | | |
| Public policies | **Tax incentives for companies** | 4.0 | 1.0 | 58.3% | **Not achieved** |
| Anonymized comments: “Useful but often ineffective without broader structural policies.” “Risk of being symbolic rather than impactful.” | | | | | |
| Research priorities | **Support networks** | 3.0 | 1.0 | 41.7% | **Not achieved** |
| Anonymized comments: “Important but needs clearer definition (family? workplace? community?).” “Support networks matter, but evidence is still limited. | | | | | |

# 3. Reassessment of Items Without Consensus

Participants were asked to reassess the following items using the five-point Likert scale: **1 = Strongly disagree; 2 = Disagree; 3 = Neutral; 4 = Agree; 5 = Strongly agree.**

| **Domain** | **Item** | **Response scale** |
| --- | --- | --- |
| Individual factors | **Formal education** | 1–5 Likert + optional comment |
| Public policies | **More protective contracts** | 1–5 Likert + optional comment |
| Public policies | **Tax incentives for companies** | 1–5 Likert + optional comment |
| Research priorities | **Support networks** | 1–5 Likert + optional comment |

# 4. Evaluation of New Items Suggested by Experts

Participants were also invited to evaluate new items proposed during Round 2. Each item was rated on the same five-point Likert scale.

| **Domain** | **New item** | **Response scale** |
| --- | --- | --- |
| Individual factors | **Meaning or purpose at work** | 1–5 Likert + optional comment |
| Individual factors | **Career adaptability** | 1–5 Likert + optional comment |
| Individual factors | **Autonomy and self-regulation** | 1–5 Likert + optional comment |
| Individual factors | **Work–life balance strategies** | 1–5 Likert + optional comment |
| Public policies | **Funding for age-friendly adaptations** | 1–5 Likert + optional comment |
| Public policies | **Recognition of prior learning** | 1–5 Likert + optional comment |
| Public policies | **Anti-ageism campaigns** | 1–5 Likert + optional comment |
| Public policies | **Policy-regulated flexible arrangements** | 1–5 Likert + optional comment |
| Public policies | **Career transition support** | 1–5 Likert + optional comment |

# 5. General Comments

Participants were given an optional open-ended field to provide general comments, justifications, or additional suggestions relevant to the study.

**Note.** This document represents the controlled quantitative feedback provided between Delphi Rounds 2 and 3. Round 1 consisted exclusively of open-ended questions; therefore, no quantitative controlled feedback was provided between Rounds 1 and 2.
